# Simplified Symptom Pattern Method for verbal autopsy analysis: multisite validation study using clinical diagnostic gold standards

**DOI:** 10.1186/1478-7954-9-30

**Published:** 2011-08-04

**Authors:** Christopher JL Murray, Spencer L James, Jeanette K Birnbaum, Michael K Freeman, Rafael Lozano, Alan D Lopez

**Affiliations:** 1 Institute for Health Metrics and Evaluation, University of Washington, 2301 Fifth Ave., Suite 600, Seattle, WA 98121, USA; 2 Department of Health Services, University of Washington, Seattle, WA, USA; 3 School of Population Health, University of Queensland, Brisbane, Australia

**Keywords:** Verbal autopsy, Symptom Pattern, validation, gold standard

## Abstract

**Background:**

Verbal autopsy can be a useful tool for generating cause of death data in data-sparse regions around the world. The Symptom Pattern (SP) Method is one promising approach to analyzing verbal autopsy data, but it has not been tested rigorously with gold standard diagnostic criteria. We propose a simplified version of SP and evaluate its performance using verbal autopsy data with accompanying true cause of death.

**Methods:**

We investigated specific parameters in SP's Bayesian framework that allow for its optimal performance in both assigning individual cause of death and in determining cause-specific mortality fractions. We evaluated these outcomes of the method separately for adult, child, and neonatal verbal autopsies in 500 different population constructs of verbal autopsy data to analyze its ability in various settings.

**Results:**

We determined that a modified, simpler version of Symptom Pattern (termed Simplified Symptom Pattern, or SSP) performs better than the previously-developed approach. Across 500 samples of verbal autopsy testing data, SSP achieves a median cause-specific mortality fraction accuracy of 0.710 for adults, 0.739 for children, and 0.751 for neonates. In individual cause of death assignment in the same testing environment, SSP achieves 45.8% chance-corrected concordance for adults, 51.5% for children, and 32.5% for neonates.

**Conclusions:**

The Simplified Symptom Pattern Method for verbal autopsy can yield reliable and reasonably accurate results for both individual cause of death assignment and for determining cause-specific mortality fractions. The method demonstrates that verbal autopsies coupled with SSP can be a useful tool for analyzing mortality patterns and determining individual cause of death from verbal autopsy data.

## Background

Methods for analyzing verbal autopsies (VAs) seek to predict causes of death and/or cause-specific mortality fractions (CSMFs) based solely on a decedent's signs and symptoms leading up to death. The signs and symptoms for a given death are recorded in an interview with a member of the decedent's family. The family member's responses can then be analyzed to deduce the true cause of death through either physician-certified verbal autopsy (PCVA) or computer-coded verbal autopsy (CCVA). One CCVA approach proposed in 2007 by Murray et al. [1] was the Symptom Pattern (SP) Method. SP is a Bayesian approach that implements statistical machinery similar to the InterVA program [2], developed by Byass et al. [3] in 2003. InterVA relies on expert judgment to determine the probability of a particular cause of death given a reported symptom, while SP is a data-driven approach which invokes 1) King-Lu direct CSMF estimation [4] as the prior probability distribution, and 2) the actual probability of responses to combinations of items conditional on true cause in verbal autopsy data, which includes the true cause of death. The validated verbal autopsy data essentially trains the model, and the resulting model can then be applied to verbal autopsy questionnaires for which the true cause of death is unknown. These unknown deaths are then assigned a predicted cause of death based on the posterior distribution of the probability of death being due to each cause. Each cause's predicted deaths can then be aggregated to produce estimates of cause-specific mortality fractions in the population of verbal autopsy data being analyzed.

The SP Method has previously been implemented in the R programming language due to its flexibility and compatibility with the King-Lu algorithm. For users unfamiliar with computer programming, this interface can pose difficulties. Furthermore, the computational complexity and depth used in both the King-Lu and SP algorithms can make it difficult for operators to unpack the quantitative rationale of a cause assignment for a particular death. Despite these obstacles, SP has demonstrated success in both assigning individual cause of death and determining cause-specific mortality fractions. In a study of verbal autopsy data from China, SP performed better than PCVA [1].

During the last four years of verbal autopsy research, a number of conceptual, methodological, and empirical innovations have occurred. First, it is increasingly clear that methods such as King-Lu and SP can identify very complex patterns in data. It is essential in evaluating these methods to strictly separate training and test data even when complex resampling is undertaken. Preventing the contamination of test data with train data permits the evaluation of how well a given method will work in practice. Second, Murray et al. [5] have identified that many metrics of performance such as specificity or relative and absolute error in CSMFs are sensitive to the CSMF composition of the test data set. Robust assessment of performance must be undertaken across a range of test datasets with widely varying CSMF compositions. Further, metrics of individual concordance need to be corrected for chance to adequately capture how well a method does over and above random or equal assignment across causes. Third, the Population Health Metrics Research Consortium (PHMRC) multisite study [6] provides the first large-scale data set where rigorous clinical diagnostic criteria have been used to assign cause of death in the validation dataset. The availability of improved gold standards provides an opportunity to assess more accurately how well methods perform.

Several developments suggest that SP as originally proposed can be simplified with enhanced performance. Flaxman et al. [7] have studied when the King-Lu method of direct CSMF estimation provides accurate CSMFs. They report that when the cause list is larger than seven to 10 causes, the results of King-Lu can be quite inaccurate. Using these CSMFs as a prior in SP may actually make performance of the method worse. Lessons learned in studies of pairwise analysis [8] have also suggested that two strategies may improve performance: 1) developing models for each cause compared to all other causes, one at a time, may be better than a model for all causes at once, and 2) using a smaller, more informative set of items for each cause may improve performance. Building on these insights, we propose a simplified version of the Symptom Pattern Method and assess its performance using the PHMRC gold standard validation train and test datasets.

## Methods

### Options for modifying the Symptom Pattern Method

The basis for the SP Method is Bayes' theorem applied to cause of death analysis. Formally:

Where *S*_*i *_is the response pattern on a set of *k *items in the VA (not simply one item), and where *P*(*D*_*i *_= *j*|*S*_*i*_) is the probability of individual *i *dying from cause *j*, conditional on the observed vector of symptom responses, *S*_*i*_. Examination of Bayes' theorem highlights four options for SP modification.

First, we can develop a model for one cause at a time that produces a posterior probability of a death being from that cause or not from that cause. In the notation provided, *D*_*i *_*= j *or not *j*. Alternatively we can develop a model as originally proposed for all causes at the same time where *D*_*i *_*= j *for *j *from 1 to the last cause.

Second, the prior can be based as originally proposed on the application of the King-Lu approach to direct CSMF estimation, or it can be based on a uniform prior where all causes are considered to be equally likely. In the case of single cause models, a uniform prior would say the probability of a death being from cause *j *and all other causes other than *j *would be equal.

Third, in the original SP the responses on all items were used simultaneously. Alternatively, we have observed in other verbal autopsy research that it is possible to improve signals in the data by only including the most informative items for a given cause in that cause-specific model. Specifically, we can use the top items for a cause ordered by their tariff [9]. Tariff is most easily viewed as a robust Z score identifying when particular signs or symptoms have high information content for a particular cause. In this analysis, we tested a range of options and conducted our comparative analyses using the top 40 items per cause in terms of the absolute value of the tariff.

Fourth, we can vary the number of items evaluated at each time to determine a response pattern. The original SP paper used 16. Here we have evaluated using a cluster size of 10 versus one. The lower cluster size of 10 compared to 16 improves speed and stability of the results without reducing performance. We have evaluated dropping all interdependencies, because a method with cluster size one could be implemented much more efficiently in many computational platforms. Understanding the importance of clustering is an important dimension to SP.

Because using the top 40 symptoms ordered by tariff is only meaningful for single cause models, in total these four options yield 12 possible modifications of SP. In all of these modifications, including the single cause models, we have assigned the final cause of death using the highest posterior value by cause. When assigning more than one cause of death, we have assigned the highest posterior first, the second highest next, etc.

### Validation using the PHMRC gold standard train-test datasets

As described elsewhere in more detail [6], the PHMRC gold standard verbal autopsy validation study provides a unique and large multisite dataset to assess the performance of new or existing verbal autopsy methods. The PHMRC study collected VAs on deaths that met defined clinical diagnostic criteria for cause of death. For example, a death from an acute myocardial infarction required evidence as obtained by one or more of the following: a cardiac perfusion scan; ECG changes; documented history of coronary artery bypass surgery, percutaneous transluminal coronary angioplasty, or stenting; coronary angiography; and/or enzyme changes in the context of myocardial ischemia. As part of the PHMRC study, all variables including free-text responses regarding health care experiences (HCE) have been converted into a series of dichotomous items, which can be analyzed by SP. Table 1 provides the number of items in the adult, child, and neonatal modules. The PHMRC has developed a fixed set of 500 train and test splits of the data to allow for direct performance comparison between methods. We have analyzed all 500 of these splits for the final validation results presented in this paper. We have used the first 100 and second 100 splits to select the best variant of SP for simplifying the approach. For each split, we use the training data for SP to establish the P(Sik|Di = j) and then apply these patterns to the test dataset. In no case are there deaths in the training data that are replicated in the test data. Further, the cause composition of the test dataset is based on a random draw from an uninformative Dirichlet distribution so that the cause composition of the training data and test data are always different.

**Table 1:** Numbers of items in adult, child, and neonate modules

	Dichotomous	Continuous	Categorical	Free text	Total
**Adult**	130	25	32	7*	194

**Child**	55	13	29	7*	104

**Neonate**	76	21	33	7*	137

*Free text responses were dichotomized as individual words and expanded into 106, 90, and 39 items for adults, children, and neonates, respectively. Total does not include these expanded items.

### Simplifying Symptom Pattern

To select the best-performing variant, we conducted three types of analyses. We assess the performance of the different variants of SP at assigning individual causes of death using median chance-corrected concordance by cause across the first 100 test datasets and the median average chance-corrected concordance across causes in the 100 test datasets following the recommendations of Murray et al. [5]. For assessing the performance of SP in estimating CSMFs, we report median CSMF accuracy [5] as well as concordance correlation coefficients by cause as a summary of the relationship between estimated CSMFs for a cause and the true CSMF in a particular test dataset. To explore the comparative performance of all 12 SP variants, we have undertaken this assessment for adults, children, and neonates using household recall of HCE. On the basis of these results, we have selected a simplified approach, which we have implemented for children and neonates. To insure that this analysis did not yield results that were biased by analyzing the first 100 train-test splits, we repeated this analysis for the second 100 splits. We also confirmed that the results were robust to the selection of splits by analyzing five sets of randomly-drawn test-train splits of size 50. In the text, we present results for the analysis of the first 100 splits, but our findings are robust across the other tests. On the basis of these results, we select one variant as the Simplified Symptom Pattern (SSP) Method.

### Validation of Simplified Symptom Pattern Method

Using the full 500 train-test splits in the PHMRC dataset, we assess the performance of the SSP Method. We benchmark variants of SP with each other and against PCVA in the same dataset using the results reported by Lozano et al. [10].

Murray et al. [1] analyzed data for China two ways: including all items and excluding items that reflected the decedent's contact with health services. The purpose of excluding the latter structured and free-text items was to assess how VA would perform in poor rural populations without access to care. They found, for example, that a considerable component of PCVA performance was related to the household recall of hospital experience or availability of a death certificate or other records from the hospital. We have assessed the performance of our SSP Method in adults, children, and neonates excluding the household recall of HCE.

## Results

### Analysis of the performance of SP alternatives

Table 2 summarizes the median chance-corrected concordance and CSMF accuracy for all 12 SP variants on each age module including household recall of HCE. The table identifies each variant in terms of four attributes: symptom cluster size (10 versus one), cause-models (models for each single cause compared to noncause versus one model for multiple causes), the number of symptoms used in the likelihood step of Bayes' theorem (all versus the top 40), and the prior CSMF distribution (based on the application of King-Lu versus a uniform prior). The best results for adults are for the variant that uses a cluster size of 10, models for each cause compared to noncause, the top 40 symptoms, and a uniform prior. However, we observed that other variants produced higher performance in children and neonates. We chose to use the model specifications that produced the most consistent results across age modules by considering the rank of each variant for each age group on both chance-corrected concordance and CSMF accuracy. In particular, we found that using a cluster size of 10, running single cause models, using all symptoms, and using a uniform prior would produce the best results across modules. A close second in terms of overall performance is the variant using a cluster size of 10, running single cause models, using the top 40 symptoms based on tariff, and using a uniform prior. In fact, this variant did best on both metrics for adults but worse for neonates and children than the variant selected. The only difference between the two top performing variants is the set of symptoms included. In general, changes from single cause models to one model for multiple causes have small decrements in performance. Large drops in performance are associated with shifting from the uniform prior to the King-Lu prior and shifting from using a symptom cluster size of 10 compared to one.

**Table 2:** Comparisons of different Symptom Pattern variants based on 100 splits for the adult, child, and neonate modules, including use of health care experience information

**Adult module**:					
**Cluster**	**Single/Multiple**	**Symptom**	**Prior**	**CSMF accuracy (95% uncertainty interval [UI])**	**Chance-corrected concordance (%) (95% UI)**

**10**	Single	Top 40	Uniform	0.726 (0.714, 0.737)	47.8 (47.4, 48.2)

**10**	Single	All	Uniform	0.703 (0.687, 0.718)	45.6 (44.9, 46.3)

**10**	Single	Top 40	King-Lu	0.653 (0.640, 0.672)	42.6 (42.1, 43.4)

**10**	Single	All	King-Lu	0.311 (0.291, 0.349)	18.4 (17.4, 20.3)

**10**	Multiple	All	Uniform	0.714 (0.697, 0.721)	46.1 (45.7, 46.5)

**10**	Multiple	All	King-Lu	0.708 (0.696, 0.719)	46.0 (45.6, 46.6)

**1**	Single	Top 40	Uniform	0.668 (0.652, 0.681)	42.7 (42.2, 43.0)

**1**	Single	All	Uniform	0.632 (0.620, 0.643)	40.3 (39.8, 40.5)

**1**	Single	Top 40	King-Lu	0.163 (0.147, 0.212)	9.3 (8.3, 11.0)

**1**	Single	All	King-Lu	0.043 (0.031, 0.057)	0.4 (0.0, 0.9)

**1**	Multiple	All	Uniform	0.651 (0.636, 0.665)	39.2 (38.4, 39.4)

**1**	Multiple	All	King-Lu	0.646 (0.630, 0.664)	38.6 (38.1, 39.2)

**Child module**:					

**Cluster**	**Single/Multiple**	**Symptom**	**Prior**	**CSMF accuracy (95% UI)**	**Chance-corrected concordance (%) (95% UI)**

**10**	Single	Top 40	Uniform	0.718 (0.699, 0.738)	45.2 (44.4, 46.2)

**10**	Single	All	Uniform	0.740 (0.727, 0.757)	50.9 (50.1, 51.8)

**10**	Single	Top 40	King-Lu	0.633 (0.617, 0.666)	40.4 (39.2, 40.9)

**10**	Single	All	King-Lu	0.469 (0.453, 0.516)	36.8 (35.4, 38.0)

**10**	Multiple	All	Uniform	0.749 (0.736, 0.766)	51.8 (50.7, 52.9)

**10**	Multiple	All	King-Lu	0.759 (0.745, 0.771)	52.1 (51.5, 53.0)

**1**	Single	Top 40	Uniform	0.696 (0.676, 0.715)	44.4 (44.0, 45.5)

**1**	Single	All	Uniform	0.705 (0.692, 0.727)	46.9 (45.6, 47.5)

**1**	Single	Top 40	King-Lu	0.263 (0.228, 0.280)	16.6 (14.2, 17.7)

**1**	Single	All	King-Lu	0.125 (0.104, 0.161)	3.6 (2.3, 4.5)

**1**	Multiple	All	Uniform	0.716 (0.701, 0.733)	47.9 (46.5, 48.7)

**1**	Multiple	All	King-Lu	0.723 (0.705, 0.741)	47.9 (47.1, 48.6)

**Neonate module**:					

**Cluster**	**Single/Multiple**	**Symptom**	**Prior**	**CSMF accuracy (95% UI)**	**Chance-corrected concordance (%) (95% UI)**

**10**	Single	Top 40	Uniform	0.748 (0.730, 0.766)	29.7 (28.7, 30.6)

**10**	Single	All	Uniform	0.741 (0.720, 0.787)	31.7 (31.2, 33.0)

**10**	Single	Top 40	King-Lu	0.679 (0.647, 0.704)	27.9 (25.9, 28.5)

**10**	Single	All	King-Lu	0.603 (0.553, 0.624)	19.1 (18.0, 21.8)

**10**	Multiple	All	Uniform	0.732 (0.712, 0.745)	34.1 (32.8, 35.5)

**10**	Multiple	All	King-Lu	0.736 (0.711, 0.752)	33.6 (32.9, 35.5)

**1**	Single	Top 40	Uniform	0.663 (0.634, 0.691)	28.8 (27.4, 29.6)

**1**	Single	All	Uniform	0.604 (0.571, 0.639)	26.4 (25.2, 27.6)

**1**	Single	Top 40	King-Lu	0.425 (0.391, 0.462)	10.0 (9.2, 11.6)

**1**	Single	All	King-Lu	0.363 (0.325, 0.384)	0.0 (0.0, 0.5)

**1**	Multiple	All	Uniform	0.564 (0.550, 0.580)	29.5 (27.7, 30.4)

**1**	Multiple	All	King-Lu	0.565 (0.541, 0.591)	29.4 (27.8, 30.8)

Our findings on which variant performs best were consistent across other tests, including reassessment of performance for the second 100 test-train splits and assessment on randomly drawn test-train splits. In all cases, the shift from uniform priors to King-Lu priors and from cluster size 10 to cluster size one is associated with substantial decrements in performance. This simplified variant of SP -Simplified Symptom Pattern - performs substantially better than the original version published in 2007.

### Simplified SP applied to adults, children, and neonates compared to PCVA

#### Individual cause assignment

Table 3 shows the comparative performance of SSP versus PCVA in terms of chance-corrected concordance. For adults, SSP outperforms PCVA on the same test datasets both with and without household recall of health care experience. For children, SSP produces better chance-corrected concordance in comparison to PCVA both when health care information is added and withheld. For neonates, SSP does better than PCVA without HCE and slightly worse than PCVA when HCE information is added, though direct comparison is not possible since PCVA analysis was limited to six neonatal causes, while SSP predicted for 11 neonatal causes.

**Table 3:** Median chance-corrected concordance (%) for SSP and PCVA, by age group with and without HCE

		SSP		PCVA	
		**Median**	**95% UI**	**Median**	**95% UI**

**Adult**	**No HCE**	38.0	(37.8, 38.1)	29.7	(29.4, 29.8)

	**HCE**	45.8	(45.7, 45.9)	44.6	(44.3, 44.8)

**Child**	**No HCE**	46.8	(46.5, 47.3)	36.3	(35.9, 36.6)

	**HCE**	51.5	(51.1, 51.9)	47.8	(47.1, 48.3)

**Neonate**	**No HCE**	30.4	(30.0, 30.7)	27.6	(27.2, 28.0)

	**HCE**	32.5	(32.0, 33.0)	33.3	(32.8, 33.7)

The median chance-corrected concordance is computed as the median across 500 splits of the mean chance-corrected concordance across causes. These results show how SSP outperforms physicians in individual cause assignment in every situation where head-to-head comparison is possible, except for the neonatal module with the HCE information added. In the neonatal module, SSP cannot be directly compared to PCVA since PCVA analysis could only be conducted for six neonate causes, while SSP can predict for 11 causes.

**Figure 1:**
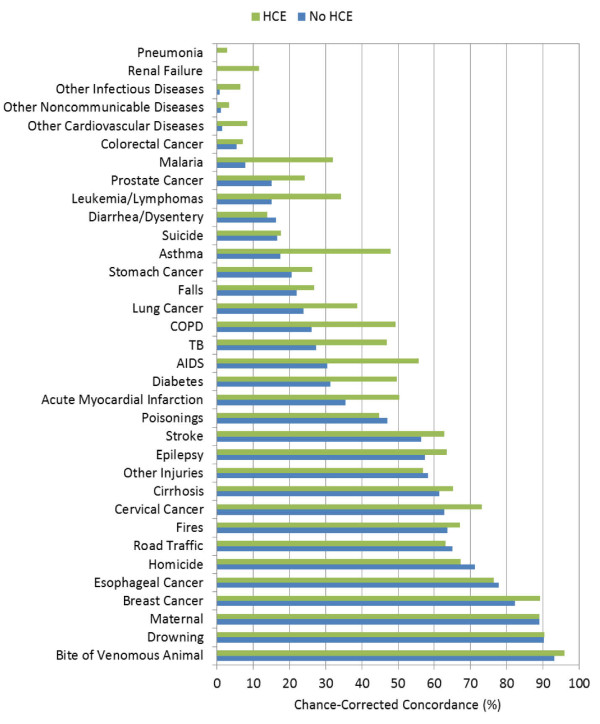
**Median chance-corrected concordance (%) across 500 Dirichlet splits, by adult cause with and without HCE**.

**Figure 2:**
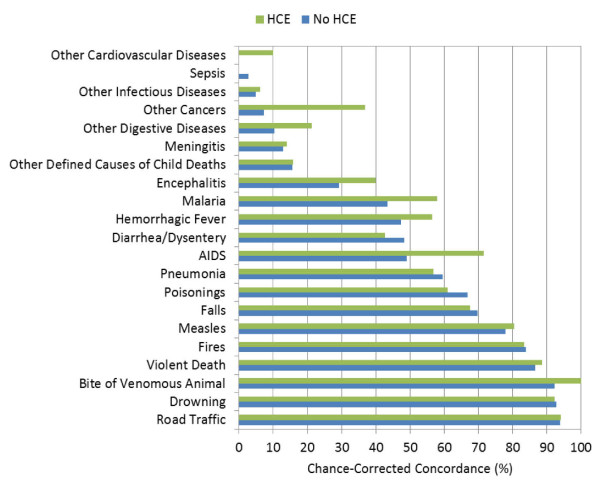
**Median chance-corrected concordance (%) across 500 Dirichlet splits, by child cause with and without HCE**.

**Figure 3:**
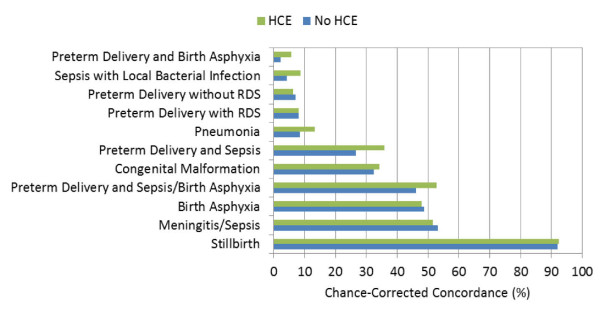
**Median chance-corrected concordance (%) across 500 Dirichlet splits, by neonate cause with and without HCE**.

Figures 1, 2, and 3 highlight the hierarchy of cause-specific chance-corrected concordances in the adult, child, and neonatal modules, respectively. These figures also emphasize the extent to which the addition of health care experience information can inform the predictions for certain causes. AIDS in the adult module, for example, achieves much higher chance-corrected concordance upon addition of HCE. Additional file 1 provides the chance-corrected concordances by cause with and without HCE for SSP. Remarkably, for 15 adult causes with HCE, chance-corrected concordances are above 50%. These causes include all the injuries but also causes such as stroke, AIDS, cirrhosis, cervical cancer, esophageal cancer, and breast cancer. Even when HCE is excluded, chance-corrected concordance is higher than 50% for 13 causes. The causes with the worst performance included some cancers such as colorectal, stomach, prostate, and leukemia/lymphoma. Residual categories such as other noncommunicable, other cardiovascular, and other infectious diseases do particularly poorly. In addition, both renal failure and pneumonia are notable for very low chance-corrected concordances.

Additional file 1 for children highlights good performance for the injuries but also for measles, hemorrhagic fever, AIDS, pneumonia, and malaria. As with adults, poor performance is notable for residual categories such as other cancers, other infectious diseases, and other cardiovascular diseases. In neonates (also shown in Additional file 1) SSP does well for stillbirths, preterm delivery and sepsis/birth asphyxia, meningitis/sepsis, and birth asphyxia.

#### CSMF estimation

Table 4 shows the CSMF accuracy achieved by SSP in comparison to PCVA for adults, children, and neonates with and without HCE. In all cases, SSP performs substantially better and generates more accurate estimated CSMFs than PCVA on exactly the same validation datasets. Neonate results for CSMF accuracy are not comparable from PCVA to SSP because the PCVA results are compiled at a six-cause level, whereas SSP is capable of producing estimates for 11 different causes. The difference in adults and children can be as large as 0.077 for children without HCE. This represents a substantial increment in performance at the population level relative to PCVA.

**Table 4:** Median CSMF accuracy for SSP and PCVA, by age group with and without HCE

		SSP		PCVA	
		**Median**	**95% UI**	**Median**	**95% UI**

**Adult**	**No HCE**	0.671	(0.664, 0.676)	0.624	(0.619, 0.631)

	**HCE**	0.710	(0.704, 0.714)	0.675	(0.669, 0.680)

**Child**	**No HCE**	0.709	(0.700, 0.717)	0.632	(0.626, 0.642)

	**HCE**	0.739	(0.733, 0.745)	0.682	(0.671, 0.690)

**Neonate**	**No HCE**	0.748	(0.736, 0.759)	0.695	(0.682, 0.705)

	**HCE**	0.751	(0.737, 0.764)	0.733	(0.719, 0.743)

To explore the variation by cause in SSP's mortality fraction estimation, we modeled the estimated CSMF as a function of true CSMF. Additional file 2 shows this relationship based on the true and estimated results from 500 different test splits in the form

**Figure 4:**
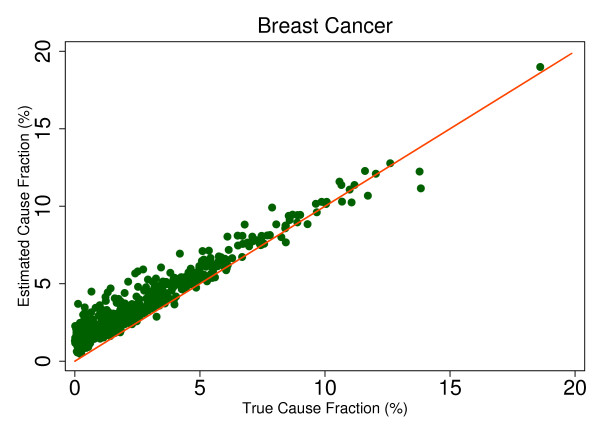
**True versus estimated mortality fractions for breast cancer, adult module with HCE information**.

**Figure 5:**
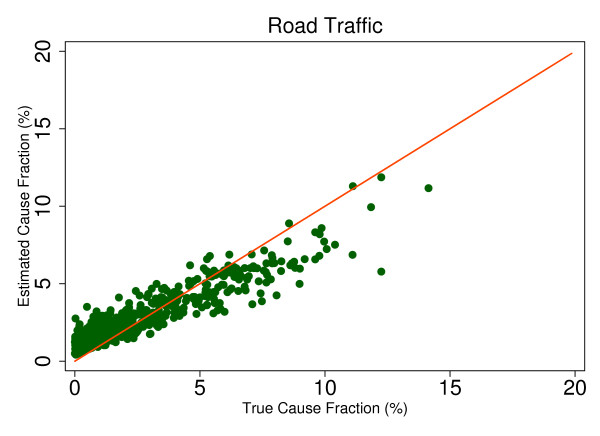
**True versus estimated mortality fractions for road traffic, adult module with HCE information**.

**Figure 6:**
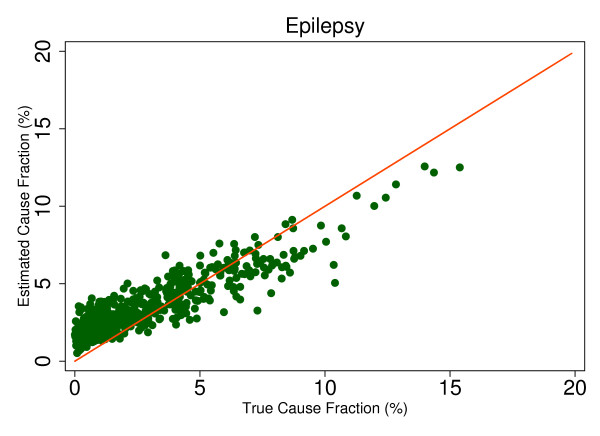
**True versus estimated mortality fractions for epilepsy, adult module with HCE information**.

**Figure 7:**
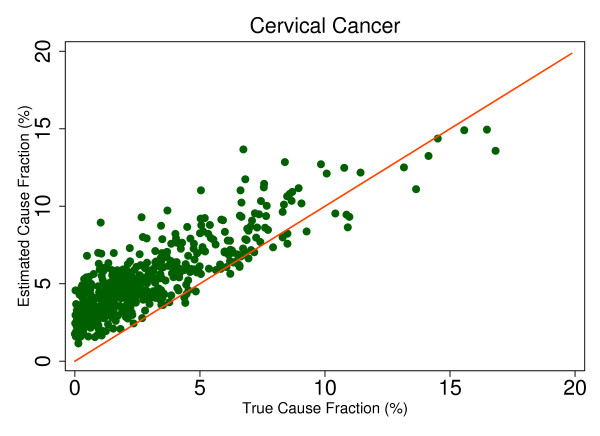
**True versus estimated mortality fractions for cervical cancer, adult module with HCE information**.

**Figure 8:**
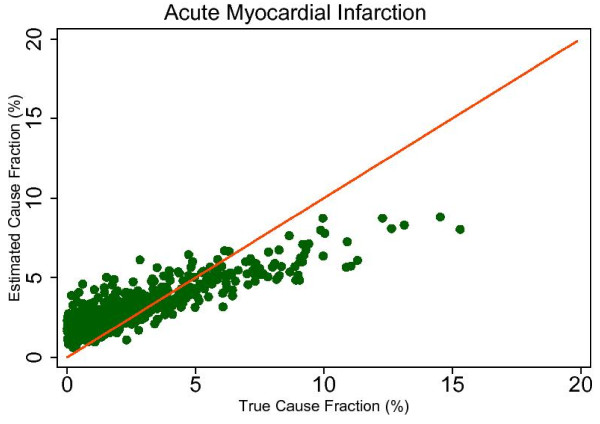
**True versus estimated mortality fractions for acute myocardial infarction, adult module with HCE information**.

**Figure 9:**
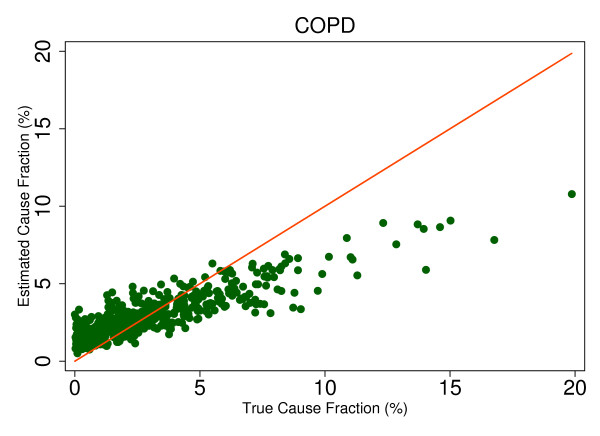
**True versus estimated mortality fractions for COPD, adult module with HCE information**.

This regression allows us to observe the predicted size of any cause's mortality fraction even if no true deaths from that cause exist in the dataset and then to determine whether SSP will tend to overestimate or underestimate if the true mortality fraction is greater than zero. Extracting the root mean square error (RMSE) allows for assessment of the range of estimated CSMFs for a given true CSMF, therefore indicating whether any over- or underestimation will be systematic and predictable. This analysis is a useful way to predict how SSP could perform in the field, particularly considering the different settings and project aims that may be focused on different disease burdens. Based on the results from this regression, we chose six causes that highlight characteristics of SSP's predictions. Figures 4, 5, 6, 7, 8 and 9 show a comparison of estimated CSMFs and true CSMFs for these six causes: breast cancer (Figure 4), road traffic (Figure 5), epilepsy (Figure 6), cervical cancer (Figure 7), acute myocardial infarction (Figure 8), and chronic obstructive pulmonary disease (COPD) (Figure 9).

Breast cancer, shown in Figure 4, exemplifies a cause for which SSP produces accurate CSMF estimates regardless of the true CSMF size. It has a tendency to slightly overestimate the CSMF when the true CSMF is very small. Indeed, results from the regression show that SSP will predict a CSMF of 1.4% even if there are no actual deaths from breast cancer. The slope of the regression in addition to the scatter show, though, that beyond very small CSMFs for breast cancer, SSP will typically produce predicted CSMFs that are very close to the truth. Road traffic in Figure 5 shows a very similar relationship. Both breast cancer and road traffic are causes that also obtain a high chance-corrected concordance, suggesting a strong relationship between success at individual-level assignment and population-level estimates. Figure 6 shows how for epilepsy, SSP will overestimate at lower true CSMFs, but as the true fraction increases, SSP begins to underestimate. The regression results confirm this observation. The intercept of the regression for epilepsy is 0.017, indicating an estimated CSMF of 1.7% will occur even if no true epilepsy deaths exist. The slope of 0.636 and the accompanying scatter both suggest that beyond a CSMF of approximately 4%, SSP will begin to systematically underestimate the mortality fraction from epilepsy. Cervical cancer, shown in Figure 7, highlights a case where SSP more dramatically overestimates the CSMF when the true CSMF is less than approximately 9%. Beyond 9%, however, the estimations tend to be closer to truth. The RMSE for the cervical cancer regression is 0.013, twice as large as the RMSE for breast cancer, indicating a noisier range of estimates for any given true CSMF. Acute myocardial infarction in Figure 8 is another cause for which SSP systematically underestimates beyond a 5% true cause fraction, and has a RMSE of 0.008. A very similar relationship is shown for COPD in Figure 9.

**Figure 10:**
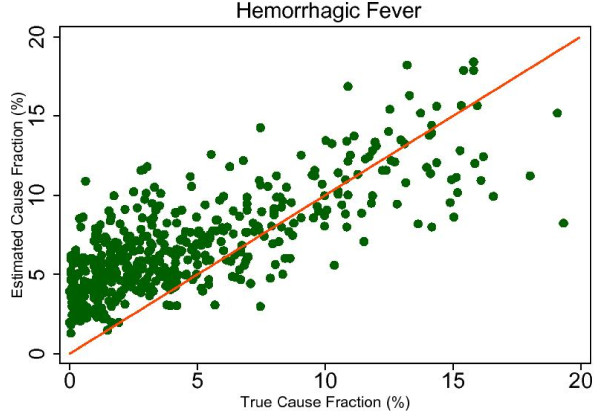
**True versus estimated mortality fractions for hemorrhagic fever, child module with HCE information**.

**Figure 11:**
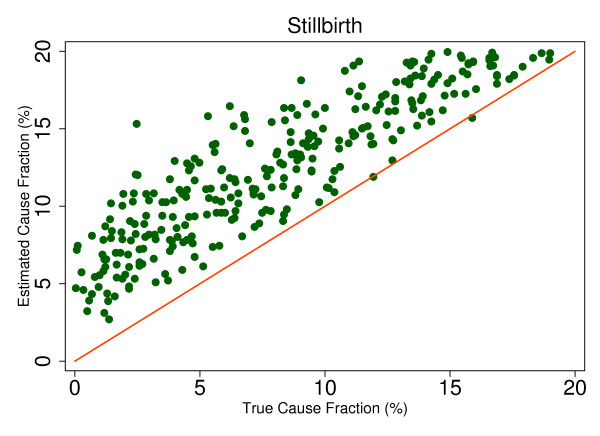
**True versus estimated mortality fractions for stillbirths, neonate module with HCE information**.

The RMSE in the adult results with HCE ranges from 0.003 to 0.015. In the child with HCE results, the RMSE is typically higher, ranging from 0.006 to 0.027, highlighting the noisier CSMF estimations that result from SSP's use with child VAs. For example, Figure 10 shows the true and estimated CSMFs for hemorrhagic fever in children, which evidently produces a range of estimates for any given true CSMF. The neonate CSMF estimation is also typically less precise than the adult results, with a RMSE ranging from 0.012 to 0.056. The true and estimated CSMFs for stillbirths are shown in Figure 11 and demonstrate a cause which is essentially always subject to overestimation by SSP. Overall, the analysis of the true versus estimated relationships suggests that while systematic underestimation or overestimation beyond a certain threshold CSMF may be an intrinsic characteristic of SSP's predictions, in many cases the trend is still predictable and precise.

## Discussion

These results suggest that Simplified Symptom Pattern performs better than the original version proposed by Murray et al. in 2007. In fact, by dropping the use of the King-Lu direct CSMFs as the prior in SSP, performance has improved. This is consistent with the finding of Flaxman et al. [7] that King-Lu has poor accuracy when there are more than seven to 10 causes in the cause list. SSP performance is also enhanced by developing models for each cause, one at a time, that predict whether a death is from a given cause compared to all other causes and then picking the cause with the highest posterior probability across the individual cause models. SSP is further improved by using a cluster size of 10. These simplifications have led to substantial improvement in performance.

Simplified Symptom Pattern performs remarkably well both at individual cause assignment and CSMF estimation. SSP has higher than or equivalent chance-corrected concordance and CSMF accuracy than PCVA in all cases, except for the chance-corrected concordance for neonates with the inclusion of HCE information. The relative differences in performance, particularly concerning CSMF accuracy, between the various implementations of PCVA and SSP presented in this paper may seem minimal. However, we have observed that incremental increases in CSMF accuracy in fact represent substantial improvements. The CSMF accuracy ranges from 0.624 to 0.751 across all the cases in this paper. Two methods would differ in CSMF accuracy by 10 percentage points if on average over 500 tests, one cause was misestimated to be 10 CSMF percentage points higher on average. For the purposes of studying population health, this difference is quite important.

Lozano et al. [2] report that InterVA, which is also based on Bayes' theorem, performs markedly worse than PCVA or the SSP Method in the same validation dataset. For individual cause assignment, SSP has a chance-corrected concordance for adults that is twice as high with similarly large increments in performance in children and neonates. The substantially improved performance of SSP in the same validation datasets can be easily understood by the same dimensions that have been tested in the simplification of the method. SSP can be transformed into InterVA by four steps: use a specific InterVA subset of symptoms, use a cluster size of one, estimate a model for all causes at once, and use expert judgment about the probability of a symptom conditional on a cause of death rather than empirical patterns observed in the training data. All of these choices actually make the performance of a Bayesian approach worse as demonstrated in this analysis. Lozano et al. [2] do in fact test SSP and show that one can reduce the performance of SSP by taking on these InterVA assumptions.

The main practical limitation of the SSP Method is that using a symptom cluster size greater than one requires any analysis of test data to sample from a large training dataset that captures the complex patterns in symptom clusters conditional on cause. This means that SSP cannot be easily delivered to a local analyst for the assessment of a single cause of death. The computational power required to implement SSP on a single-death basis is greater than other methods, such as the Tariff Method or Random Forest Method. For analysis of large groups of deaths or for research studies, this computational power may be a reasonable trade-off given the reliable results produced by the Simplified Symptom Pattern Method. The SSP code will be trained on the full PHMRC dataset and the model will be available for use on the Internet following publication of this paper.

## Conclusions

First developed in 2007, the Symptom Pattern Method for verbal autopsy has been subject to in-depth investigation and experimentation. The application of Bayes' theorem to verbal autopsy responses is an intuitive approach from a statistical standpoint; however, the method may be difficult to fully comprehend by some users. Consequently, it is important for the method to be implemented on a user-friendly computational platform with the option to work with different verbal autopsy instruments. In such a setting, the Simplified Symptom Pattern Method presented in this paper can produce reliable, accurate results for both individual cause of death assignment as well as cause-specific mortality fraction estimates. The growing demand for more comprehensive cause of death data in settings without functioning health information systems could be met by further implementation of verbal autopsy surveys and the use of the Simplified Symptom Pattern Method to analyze the results.

## Abbreviations

CCVA: computer-coded verbal autopsy; CSMF: cause-specific mortality fraction; HCE: health care experience; PCVA: physician-certified verbal autopsy; PHMRC: Population Health Metrics Research Consortium; RMSE: root mean square error; SP: Symptom Pattern; SSP: Simplified Symptom Pattern; VA: verbal autopsy

## Competing interests

The authors declare that they have no competing interests.

## Authors' contributions

CJLM, JKB, and SLJ conceptualized the method and algorithm. SLJ performed analyses and helped write the manuscript. MKF produced testing data. RL and ADL guided the study design and paper writing. CJLM drafted the manuscript and approved the final version. CJLM accepts full responsibility for the work and the conduct of the study, had access to the data, and controlled the decision to publish. All authors have read and approved the final manuscript.

## Supplementary Material

Additional file 1 **Median chance-corrected concordance (%) across 500 Dirichlet splits, by age group and cause with and without HCE**. Click here for file

Additional file 2 **Slope, intercept, and RMSE from linear regression of estimated versus true CSMFs, by age group and cause with and without HCE**. Click here for file
